# Factors predictive of alcohol use during pregnancy in three rural states

**DOI:** 10.1186/1744-9081-3-8

**Published:** 2007-02-09

**Authors:** Gary R Leonardson, Roland Loudenburg, Judy Struck

**Affiliations:** 1 Mountain Plains Research, 55 Rodeo Trail, Dillion, MT 59725, USA; 2 Center for Disabilities, Department of Pediatrics, Sanford School of Medicine, The University of South Dakota, P. O. Box 530, Salem, SD 57058, USA; 3 Center for Disabilities, Department of Pediatrics, Sanford School of Medicine, The University of South Dakota, 1400 West 22^nd ^Street, Sioux Falls, SD 57374, USA

## Abstract

**Background:**

A substance use screening instrument was used to determine factors predictive of drinking during pregnancy. Alcohol consumption during pregnancy can lead to negative birth outcomes.

**Methods:**

The participants (n = 4,828) for the study were sampled from pregnant women attending prenatal clinics in Montana, South Dakota, and North Dakota. Clinic sites for the administration of the screening instrument were selected in each state, based on geographic and known population characteristics. Univariate and multivariate statistical procedures were used to determine factors predictive of drinking during pregnancy.

**Results:**

Women who drank tended to: be single, be between 21–25 years old, have had fewer children, have had abortions, and be unemployed. Demographic factors that were protective of drinking when pregnant were married and full-time housewife status. Other variables associated with maternal alcohol use were: past sexual abuse, current or past physical abuse, tobacco use, other drug use, lived with substance users, and had mates who were substance users. Other contributing factors for alcohol use included: feeling sad, believing that drinking any amount of alcohol while pregnant was acceptable, had been in treatment, could use treatment now, and were able to hold four or more drinks.

**Conclusion:**

Because drinking rates were high and factors correlated with drinking are known, alcohol screening for this population is essential.

## Background

Alcohol is the most commonly used teratogen in the western world [1]. Of all the substances (e.g., cocaine, heroin, marijuana, etc.) of abuse, alcohol produces the most serious neurobehavioral effects to the fetus [2]. Prenatal exposure to alcohol is the leading preventable cause of mental retardation in the United States [3]. Additionally, one of the major health objects for Healthy People 2010 is to reduce maternal alcohol use during pregnancy to 6 percent [4].

Screening for alcohol use among pregnant women is becoming increasingly important in view of new studies that show that even low levels of prenatal alcohol exposure can have deleterious impacts on the fetus [5]. Even though approximately 20 percent of the pregnant women drink at some point in the pregnancy, maternal drinking can be difficult to measure and detect [5].

It is estimated that about 60 percent of adult American women drink at least occasionally [2]. Most women are light to moderate drinkers who have few problems related to their drinking [2]. In a national study of women conducted in 1991 [6] it was found that 44 percent were considered light drinkers, 12 percent were moderate drinkers, and 3 percent were classified as heavy drinkers. In the same study, 73 percent of the women in their 20s and 69 percent in their 30s used alcohol in the past year. Drinking rates were the highest among young women and tended to decline with age [2]. The proportion of women drinking increased between the 1940s and the 1980s [7]. The drinking rates among women have been relatively stable with slight increases in the 1970s and modest declines since the 1980s [6,8,9] along with slight declines noted for women aged 18–44 in the 1990s [10].

Estimates on the rate of pregnant women drinking ranged from 13.2 percent to over 50 percent [11-16]. The Institute of Medicine (IOM) suggests that approximately 20 percent of pregnant women drink at least some alcohol [2]. However, the majority of the children born to these women have no deleterious damage [2].

Women who have had a previous child with Fetal Alcohol Spectrum Disorders (FASD) are at high risk for drinking during subsequent pregnancies [17]. Alcohol use before pregnancy is another risk factor for alcohol use during pregnancy [10,18,19]. Marital status is a significant predictor of drinking during pregnancy. Single women have a greater likelihood of drinking during pregnancy than do married persons [14]. Married women may be less likely to use alcohol during pregnancy because of the following protective factors: superior social and financial support, and greater possibility of wanted pregnancies [14,18,20]. Smoking is a risk factor [14-16,18] for maternal substance use, as is having experienced physical and sexual abuse [14,19,21,22]. Partner's use and/or drinking by the woman's mother [2,14,19] have been correlated with maternal drinking. Additional correlates of drinking during pregnancy include limited or no transportation [14], and any reported past physical or emotional abuse [14]. The purpose of this study was to examine risk factors for drinking during pregnancies for women living in three rural states.

## Methods

### Questionnaire

The Prenatal Questionnaire (PNQ) was adapted from the Self-Administered Questionnaire (SAQ) developed within the Indian Health Service in 1997 [14] to meet the special needs of the Northern Plains Indians. The 36 items included in the PNQ queries the extent of alcohol use before and during pregnancy, along with the potential risk factors and demographic information. The psychometric properties of the SAQ (e.g., sensitivity = 76.6, specificity = 92.8, positive predictive value = 92.4) were found to be adequate [14,22]. The PNQ includes fifteen dichotomous risk factor items, based on past research and items from the original questionnaire [14,22], along with modified TACE and TWEAK questions. The changes made in the SAQ for the PNQ were minimal and it is believed that the psychometric properties would be similar, since the drinking questions are the same. We are currently validating the PNQ in another study.

Drinking on the PNQ was assessed in two ways: 1) by asking the amount and frequency of drinking during this pregnancy; and, 2) by comparing time of last drink with normal menstrual cycle as per the validation of the SAQ [22]. Physical abuse was assessed by the question, "Has anyone physically abused (hit, kicked, slapped, etc.) you during the last year." Sexual abuse was measured by the question, "Have you ever had sex without giving your consent?" The amount of alcohol a person can 'hold' was assessed by two questions: 1) "If you drink, how many drinks can you hold?" and, 2) "If you drink, how many drinks can you drink before passing out or falling asleep?"

### Sampling strategy

Medical clinics providing prenatal care were selected to participate in the study based on sampling procedures and willingness to participate in the survey. The sampling design for each state was stratified, based on geographic area or population densities and types (e.g., urban, rural, frontier, reservation). Clinics were randomly selected within these stratifications. Clinic personnel collected the completed questionnaires and returned them to the Center for Disabilities at the University of South Dakota.

Women accessing prenatal care were asked to complete the PNQ anonymously by nurses or receptionists at their first prenatal care visit. All the women completing the surveys were pregnant, although it was not known if the pregnancies were planned. If the pregnant persons decided to participate, they were given packets containing general information on the study and contact information in the event that they would like more information, along with the PNQ, and an unsealed envelope. The women were instructed to read the general information sheet and instructions, complete the PNQ, seal the completed PNQ in the envelope, and return it to the nurses or receptionists. The pregnant women were given small incentive gift certificates upon finishing the questionnaires. Since participation was voluntary and 99 sites participated, it was not possible to assess exact response or refusal rates, although state supervisors estimated that more than 90 percent of the prenatal patients participated. IRB approval for the project was obtained from the University of North Dakota, the University of South Dakota, Montana State University, and the Indian Health Service Area Office in Aberdeen, South Dakota.

## Results

### Basic demographic information

The information presented in this study was based on 4,828 questionnaires, collected at the first prenatal visit. Most (58.5%) of the respondents were in their first trimester, some (26.2%) in the second, and a few (15.2%) in their third trimester. The surveys were collected from sites in North Dakota (n = 1,688), South Dakota (n = 1,638), and Montana (n = 1,502). The average age of the pregnant women completing the PNQ was 26.7 (*sd *= 5.8) years with age ranging from 14 to 47. Women completing the PNQ averaged 13.4 (*sd *= 2.5) years of education. The women had a mean of 1.1 (*sd *= 1.1) children with responses ranging from zero to fifteen. The survey participants reported an average of 1.7 (*sd *= 1.7) pregnancies, which was consistent across states. Of those responding, 8.6 percent had a previous abortion and 24.6 percent reported a miscarriage from a prior pregnancy.

As would be expected based on population values for these three rural states, a vast majority (88.7%) of the respondents were 'White,' and 8.9 percent were Native Americans (Table 1). The total percent by 'Race/Ethnicity' equals more than 100 percent because respondents were able to select multiple 'Race/Ethnicity' categories. Most (65.7%) were 'Married,' some were 'Single' (19.0%) or 'Living Together' (13.4%), and a few (2.7%) were 'Divorced' or 'Separated.' Some persons had multiple responses to these categories. Also, a majority (69.4%) of survey participants declared themselves as employed with 15.8 percent indicating 'A full-time house wife and not looking for employment,' and 10.4 percent identified themselves as unemployed. Finally, more than one-half (54.1%) reported total family income over $30,000, and some (14.7%) reported annual family incomes less than $10,000.

**Table 1:** Demographic Factors: Overall and by State – Frequencies

	**Overall** **N = 4828**	**MT** **N = 1502**	**ND** **N = 1688**	**SD** **N = 1638**
**Race/Ethnicity**				
American Indian	8.9%	12.4%	7.9%	6.7%
Asian	0.8%	0.4%	1.0%	0.9%
Black	0.5%	0.1%	0.6%	0.7%
Pacific Islander	0.4%	0.4%	0.3%	0.4%
White	88.7%	86.3%	88.9%	90.7%
Hispanic	3.2%	4.3%	3.0%	2.4%
Other	1.1%	1.6%	0.8%	0.8%
				
**Marital Status**				
Single	19.0%	20.0%	17.1%	20.1%
Married	65.7%	59.3%	71.7%	65.4%
Living together	13.4%	18.3%	10.0%	12.5%
Separated	1.3%	2.1%	0.7%	1.1%
Divorced	1.4%	1.6%	1.2%	1.6%
				
**Employment**				
Unemployed	10.4%	14.2%	9.1%	8.2%
Full-time housewife	15.8%	18.9%	15.5%	13.2%
Employed	69.4%	61.7%	70.7%	75.0%
				
**Income**				
$0–$10,000	14.7%	20.7%	10.5%	13.7%
$10,001–$20,000	15.5%	20.2%	13.1%	13.8%
$20,001–$30,000	15.7%	16.9%	14.4%	16.0%
$30,001–$50,000	27.0%	23.1%	28.7%	28.7%
Over $50,000	27.1%	19.0%	33.3%	27.7%
				
**Overall Health**				
Excellent	15.6%	15.1%	16.5%	15.1%
Very good	45.1%	41.2%	49.5%	44.1%
Good	36.3%	39.4%	32.0%	37.9%
Fair	2.9%	4.1%	2.0%	2.8%
Poor	0.1%	0.2%	0.0%	0.2%
				
**Felt Sad, Discouraged, Hopeless, etc., in Last Month**				
Extremely	1.0%	1.6%	0.7%	0.8%
Very much	2.6%	4.1%	1.6%	2.2%
Quite a bit	4.4%	5.0%	3.2%	5.1%
Some	10.9%	13.9%	9.0%	10.1%
A little bit	26.5%	25.6%	26.2%	27.8%
Not at all	54.6%	49.8%	59.3%	54.0%

Nearly all who responded to the health question felt that they had 'Good', 'Very Good' or 'Excellent' health. Almost one-half (45.1%) of those completing the survey indicated that their health was 'Very Good' and 3.0 percent reported their health status as 'Fair' or 'Poor.' About one-fifth (18.9%) reported at least some feelings of discouragement and hopelessness in the last month. Most of the respondents from each state reported that in the last month they did not have feelings of sadness, hopelessness, discouragement, or had so many problems that they wondered if everything was worthwhile.

### Significant dichotomous factors – univariate analysis

The significance of factors predictive of drinking was determined by univariate and multivariate factors with chi square and logistic procedures, respectively. Univariate statistics were used to determine the total number of significant factors, and multivariate procedures were used to determine a smaller set of questions that retain predictive validity. Probability values equal to or less than .05 were considered statistically significant. Probabilities were adjusted, as noted, to accommodate multiple comparisons.

Based on all persons in the sample (n = 4,828), some demographic factors were found statistically significantly related to drinking status, although group differences were modest and have limited clinical utility. The drinkers had more abortions (means were .16 and .09, *t *= -5.03, *p *= .001) and fewer children (means were .94 and 1.09, *t *= 3.62, *p *= .001) than the comparison (non-drinking) group.

Income was significantly (*χ*^2 ^= 16.2, *p *= .03) related to drinking classification. In examining the percent drinking by income category, it was noted that those with low ($0–$20,000) income and high income ($50,000 or more) had the highest drinking rates. Also, survey respondents who were younger (ages 21–25) were more likely to drink than were all other age groups (*χ*^2 ^= 14.67, *p *< .001). Unemployment (*χ*^2 ^= 9.3, *p *< .04) was another demographic factor predictive of drinking status.

Factors found to be protective of drinking included: married (*χ*^2 ^= 26.1, *p *< .001), full-time housewife status (*χ*^2 ^= 27.4, *p *< 001), and employed (*χ*^2 ^= 9.3, *p *<.002). Married women had the lowest drinking rate (*χ*^2 ^= 26.1, *p *< .001) of the marital status categories, and the single women had the highest (*χ*^2 ^= 15.3, *p *< .001) rate, suggesting that marriage is a protective factor for maternal substance use and being single a risk factor. Sexual abuse (*χ*^2 ^= 27.1, *p *< .001) and physical abuse in the past year (*χ*^2 ^= 35.3, *p *< .001) were significant risk factors. Amount of alcohol consumption was another category of risk factors. Women, who believed any amount of alcohol was safe for pregnant women to drink (*χ*^2 ^= 67.7, *p *< .001) or felt that they could hold more than four drinks (*χ*^2 ^= 169.0, *p *< .001), had elevated risk levels.

The concurrent and past use of other drugs (*χ*^2 ^= 255.7, *p *< .001) and tobacco (*χ*^2 ^= 31.2, *p *< .001) were related to drinking or risk status. Additionally, being around others who used substances was correlated with risk status. Partners or mates using substances (*χ*^2 ^= 172.9, *p *< .001) and persons in household drinking or using drugs (*χ*^2 ^= 100.3, *p *< .001) were two factors related to drinking when pregnant. If partner or mate substance use was viewed as a problem by the pregnant women, the drinking risk was increased (*χ*^2 ^= 4.4, *p *= .04).

Other factors found to be predictive of drinking status were: being sad or discouraged in the last month (*χ*^2 ^= 32.1, *p *< .001), could use treatment at present time (*χ*^2 ^= 15.0, *p *< .001), and had previous participation in substance abuse treatment programs (*χ*^2 ^= 22.2, *p *< .001).

### Multivariate analysis

Logistic regression was used to assess the significance of the dichotomous demographic and social-psychological related risk factors in predicting drinking and to establish a smaller number of questions, using a backward stepwise procedure (e.g., backward likelihood ratio). Significant factors were determined for blocks (i.e., demographic, social-psychological, and past substance use and TACE-type questions) of related questions. The significant factors for each block were used in the final regression analysis phase. With this procedure, only factors that added predictive information were included in the final regression model. In the final logistic regression model, eleven factors (Table 2) were retained. These demographic, social-psychological and related factors accounted for a modest amount (*Nagelkerke R Square *= .13) of the predictability of the binary dependent measure, indicating that there are many other psychological, social, or demographic factors that are related to substance use during pregnancy.

**Table 2:** Logistic Regression Results-Risk Factors

**Significant Factors Multivariate Analysis**	**B Coefficient**	**Wald Statistic**	**Probability**
Ever had an abortion	-.48	15.95	.001
Full-time housewife	.28	6.34	.01
Physical abuse in last year	-.63	9.40	.002
Physical abuse this pregnancy	.93	7.61	.006
How many drinks can you hold	-.43	30.00	.001
Need to cut down on drinking	-.85	31.85	.001
Don't remember events	-.37	12.56	.001
Partner uses	-.67	76.82	.001
Safely drink	-.76	30.09	.001
Past drug use	-.26	7.45	.006

The final factors in the logistic regression model were: previous abortion, full-time housewife, physical abuse, perception of how many drinks a person can hold, need to cut down on drinking, mate was user of alcohol, used drugs before this pregnancy, believed that some alcohol use while pregnant was acceptable, and had experienced difficulty remembering events after drinking.

## Discussion

This study examined risk factors for maternal drinking, using a prenatal questionnaire that included substance-screening questions with a large sample of pregnant women. The samples were systematically drawn from medical clinics from three states with large geographic (e.g., rural, frontier) areas, along with some urban sites. Pregnant women were invited to complete the questionnaire anonymously during the first prenatal visit. The study results indicate that there are a number of risk factors present. The identification of the risk factors is paramount in targeting high-risk women for appropriate intervention programs.

Marital status was related to risk status. Consistent with other studies [14,16], being single was found to be a risk factor for alcohol use during pregnancy. Being married and a full-time housewife were protective factors in this study. It may be that these two later factors are indicative of a stable home environment with adequate social and economic support. Social support has been found to be an important ingredient in reducing or curtailing alcohol use in women [21].

Physical and sexual abuses were identified as risk factors for maternal alcohol use. These findings are consistent with other research [2,14,19,21]. Our study indicated that about 19 percent of those drinking had a history of sexual or physical abuse. Abuse questions need to be asked in a sensitive, non-obtrusive manner. A self-administered instrument, like the PNQ, may provide such an avenue.

A general area with potential opportunity for the amelioration of risk factors is the amount of alcohol that individuals reported drinking. Pregnant women, indicating any daily alcohol use (question: "How much alcohol can a pregnant woman safely drink each day?"), were at risk for maternal alcohol use. Nearly one-half (47.6%) of those believing that any amount of daily drinking was acceptable were found to be drinking. Additionally, many (38.0%) survey participants who felt they are able to hold 4 or more drinks were drinking during this pregnancy. Education and brief intervention programs that stress zero drinking, while pregnant, and limiting the number of drinks per occasion, while non-pregnant, could result in reduced drinking behavior and better birth outcomes.

## Limitations

This article did not discuss the overall drinking rate, which was higher than many reported results, because the topic will be addressed in another paper. Refusal rates at the clinics were unknown, because participants self-selected themselves and the state project directors did not have direct oversight of the clinics or the patients. The participants should have been asked if the pregnancy was planned. Physical and sexual abuse questions were asked as general questions without much specificity. A validation of a short version of the PNQ is currently being conducted by the FAS Consortium.

## Conclusion

Knowing the risk factors for women who will likely use alcohol during pregnancy is vitally important to health care providers so that intervention procedures can be targeted for the appropriate persons. The use of an instrument like the PNQ is essential in screening women at prenatal clinics so that the immediate attention can be given to women who are drinking. One of the goals of the regression analysis was to establish fewer questions while maintaining predictive validity.

The screening instrument should be administered as early as possible in the pregnancy to maximize the timing at which counseling or other intervention could be implemented. Once the extent and severity of alcohol use or misuse has been determined, the Institute of Medicine recommends that the clients be treated or managed with brief intervention by trained personnel [2]. A number of controlled studies have demonstrated the viability of brief intervention with pregnant women [23].

Pregnancy offers a unique opportunity to positively impact women's lives, resulting in the reduction of harmful substances such as alcohol, cigarettes, and other drugs. There is evidence that pregnancies provide increased motivation to reduce or eliminate unhealthy habits such as smoking and drinking [24].

Prevention programs for pregnant women who are drinking should consider providing a range of services such as, transportation, brief intervention, counseling, social support, prenatal classes, parenting and family functioning classes, and mental health services. A promising intervention program that targets multiple domains was developed for pregnant women by other programs included in this Center for Substance Abuse Prevention (CSAP) grant [25]. Programs that provide support to prenatal patients, who are drinking, need to be prioritized and implemented [14].

## Competing interests

The author(s) declare that they have no competing interests.

## Authors' contributions

GL and RL developed the design of the study and analysis and interpretation of data.

RL and JS were involved in the acquisition of the data.

All authors were involved in drafting the manuscript and have given final approval of the version to be published.
